# Chemical Hypoxia Brings to Light Altered Autocrine Sphingosine-1-Phosphate Signalling in Rheumatoid Arthritis Synovial Fibroblasts

**DOI:** 10.1155/2015/436525

**Published:** 2015-10-18

**Authors:** Chenqi Zhao, Uriel Moreno-Nieves, John A. Di Battista, Maria J. Fernandes, Mohamed Touaibia, Sylvain G. Bourgoin

**Affiliations:** ^1^Division of Infectious Diseases and Immunology, CHU de Quebec Research Center and Faculty of Medicine, Laval University, Quebec, QC, Canada G1V 4G2; ^2^Division of Rheumatology and Clinical Immunology, Royal Victoria Hospital, McGill University, Montreal, QC, Canada H3A 1A1; ^3^Department of Chemistry and Biochemistry, University of Moncton, Moncton, NB, Canada E1A 3E9

## Abstract

Emerging evidence suggests a role for sphingosine-1-phosphate (S1P) in various aspects of rheumatoid arthritis (RA) pathogenesis. In this study we compared the effect of chemical hypoxia induced by cobalt chloride (CoCl_2_) on the expression of S1P metabolic enzymes and cytokine/chemokine secretion in normal fibroblast-like synoviocytes (FLS) and RAFLS. RAFLS incubated with CoCl_2_, but not S1P, produced less IL-8 and MCP-1 than normal FLS. Furthermore, incubation with the S1P_2_ and S1P_3_ receptor antagonists, JTE-013 and CAY10444, reduced CoCl_2_-mediated chemokine production in normal FLS but not in RAFLS. RAFLS showed lower levels of intracellular S1P and enhanced mRNA expression of S1P phosphatase 1 (SGPP1) and S1P lyase (SPL), the enzymes that are involved in intracellular S1P degradation, when compared to normal FLS. Incubation with CoCl_2_ decreased SGPP1 mRNA and protein and SPL mRNA as well. Inhibition of SPL enhanced CoCl_2_-mediated cytokine/chemokine release and restored autocrine activation of S1P_2_ and S1P_3_ receptors in RAFLS. The results suggest that the sphingolipid pathway regulating the intracellular levels of S1P is dysregulated in RAFLS and has a significant impact on cell autocrine activation by S1P. Altered sphingolipid metabolism in FLS from patients with advanced RA raises the issue of synovial cell burnout due to chronic inflammation.

## 1. Introduction

Rheumatoid arthritis (RA) is a chronic systemic disorder that causes destruction of joints through inflammation and proliferation of the synovial membrane [1, 2]. In RA, the synovial tissue lining the joints becomes inflamed. In comparison with the normal synovial membrane, which is normally 1-2 cell layers thick, RA synovial tissue is hypertrophic and invaded by an excess of various leukocytes including neutrophils, T cells, macrophages, and monocytes [3]. This recruitment of leukocytes is likely to be mediated by selective chemotactic factors, such as interleukin-8 (IL-8) that recruits neutrophils and T cells, and monocyte chemotactic protein-1 (MCP-1) that recruits monocytes, into the synovium [4, 5]. A role for IL-8 [6, 7] and MCP-1 [8, 9] in these processes has been highlighted. The synthesis of chemokines in RA may be dependent, at least in part, on the production of inflammatory cytokines, such as IL-1*β* and tumor necrosis factor-*α* (TNF-*α*) [4], by the hypertrophic synovium and activated leukocytes. The complex cascade of production of chemokines, cytokines, and tissue-remodelling enzymes associated with leukocyte recruitment plays a role in synovial cell proliferation and joint erosion in RA [1, 2, 10]. Eventually, the thickened synovial membrane decreases capillary density and the oxygen tension in the joint [11–13]. Severe reduction of mean oxygen pressure in the RA synovium compared to that of healthy joints correlates with severity of inflammation [14–16]. The hypoxic RA joint environment in turn affects a host of genes involved in angiogenesis, apoptosis, cellular metabolism, matrix degradation, and inflammation [17]. Hypoxia drives vascular endothelial growth factor (VEGF) expression leading to angiogenesis [18–20]. The expression of cyclooxygenase-2 (COX-2) [21], matrix metalloproteinases (MMPs) [22], stromal cell-derived factor 1 [23], IL-6 and IL-8 [22, 24], and migration [25, 26] and proliferation of synovial fibroblasts as well [27], are exacerbated in response to hypoxia.

Sphingosine-1-phosphate (S1P) is a bioactive sphingolipid implicated in various pathological processes through binding to and activation of five G protein-coupled receptors designated as S1P_1*–*5_ [28]. Intracellular S1P is transported outside cells and gains access to cognate receptors for autocrine or paracrine signalling [28, 29]. The steady state level of intracellular S1P is regulated through synthesis by two sphingosine kinases (SphK1 and SphK2) and degradation either via dephosphorylation by S1P phosphatases (SGPP1 and SGPP2) or irreversible cleavage by S1P lyase (SPL) [30]. Moreover, S1P exported outside cells is dephosphorylated back to sphingosine by lipid phosphate phosphatases (LPPs), thereby attenuating its effects on the activation of surface receptors [31]. Alteration in the enzymes involved in S1P synthesis and catabolism may mediate many pathological states including arthritis (reviewed in [28, 32]).

Fibroblast-like synoviocytes from RA patients (RAFLS) express S1P_1_, S1P_2_, and S1P_3_ receptors [33]. RAFLS stimulation with S1P promotes the synthesis of cytokines/chemokines, COX-2 expression and release of prostaglandin E2 (PGE2), and cell migration, proliferation, and survival as well [33, 34]. SphK activation and high S1P levels have been reported in the synovium and synovial fluids of patients with RA [34–36]. Studies suggest a role for S1P in the pathophysiology of RA since SphK1 deficiency and blockade of S1P_1_ receptors attenuate collagen-induced arthritis in mice [37, 38]. Though SphKs can be activated by TNF-*α* and IL-1*β* to generate S1P, new evidence suggests a potential link between S1P and hypoxia in cancer and cardiovascular diseases [39, 40]. In this study we evaluated the impact of chemical hypoxia induced by CoCl_2_ on chemokine synthesis by normal FLS and RAFLS. We report that the blockade of S1P_2_ or S1P_3_ receptors attenuates CoCl_2_-mediated IL-8 and MCP-1 secretion in normal FLS but not in RAFLS. Furthermore, we provide evidence that low levels of intracellular S1P in RAFLS attenuate the S1P_2_ and S1P_3_ receptor-dependent synthesis of chemokines under conditions of chemical hypoxia.

## 2. Materials and Methods

### 2.1. Reagents

Cobalt chloride (CoCl_2_) was from Sigma Aldrich (Oakville, ON, Canada). S1P was purchased from Biomol (Plymouth Meeting, PA, USA). Human IL-8 and MCP-1 ELISA (Enzyme-Linked Immunosorbent Assay) kits were purchased from BioSource International Inc. (Camarillo, CA, USA) and R&D Systems (Minneapolis, MN, USA), respectively. The S1P_2_ and S1P_3_ receptor antagonists (JTE-013 and CAY10444) were from Cayman Chemical (Ann Arbor, MI, USA). The S1P assay kit was from Echelon Biosciences (Salt Lake City, UT, USA). SYBR Green JumpStart Ready Mix kits were obtained from Sigma (Oakville, ON, Canada). TRIzol reagent and Superscript II were purchased from Life Technologies (Burlington, ON, Canada). Anti-SGPP1 and SPL antibodies were from Novus Biologicals (Oakville, ON, Canada) and R&D Systems (Minneapolis, MN, USA), respectively. Anti-PI3 kinase p85 (06-195) was purchased from Upstate Biotechnology Associates (Billerica, MA, USA). The Proteome Profiler Human Cytokine Array (panel A) was bought from R&D Systems (Minneapolis, MN, USA). Cell culture reagents were from Wisent Inc. (St-Bruno, QC, Canada).

### 2.2. Synthesis of SPL Inhibitor

Starting chemicals and solvents were purchased from Sigma Aldrich (Oakville, ON, Canada) and Alfa Aesar (Ward Hill, MA, USA). A Biotage initiator system was used for microwave heating. Nuclear magnetic resonance (NMR) spectra were collected on a Bruker Avance III 400 MHz spectrometer with chemical shifts referenced to residual solvent peaks as secondary reference for ^1^H and ^13^C spectra. Crude products were purified using a Sg100c (Teledyne Isco) flash chromatographic instrument.

**Scheme 1:**
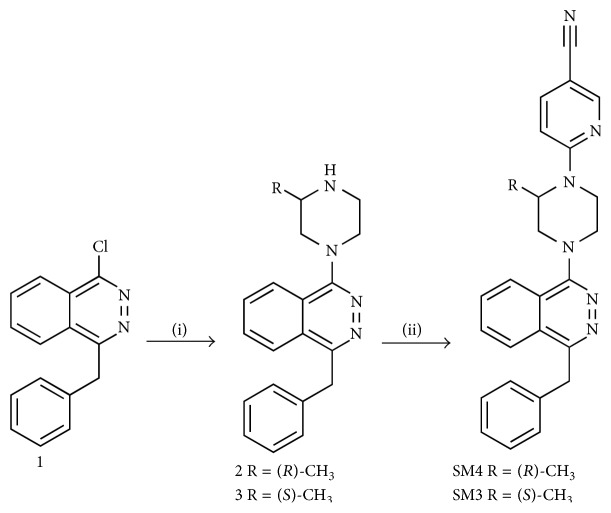
Reagents and conditions: (i) (2): Na_2_CO_3_, (*R*)-methylpiperazine, dioxane, 100°C, 48 h, 91%; (3): Na_2_CO_3_, (*S*)-methylpiperazine, dioxane, 100°C, 48 h, 88%; (ii): Na_2_CO_3_, 6-chloronicotinonitrile, DMF/dioxane, 180°C, microwave (SM4: 11%, SM3: 16%).

**Figure 1:**
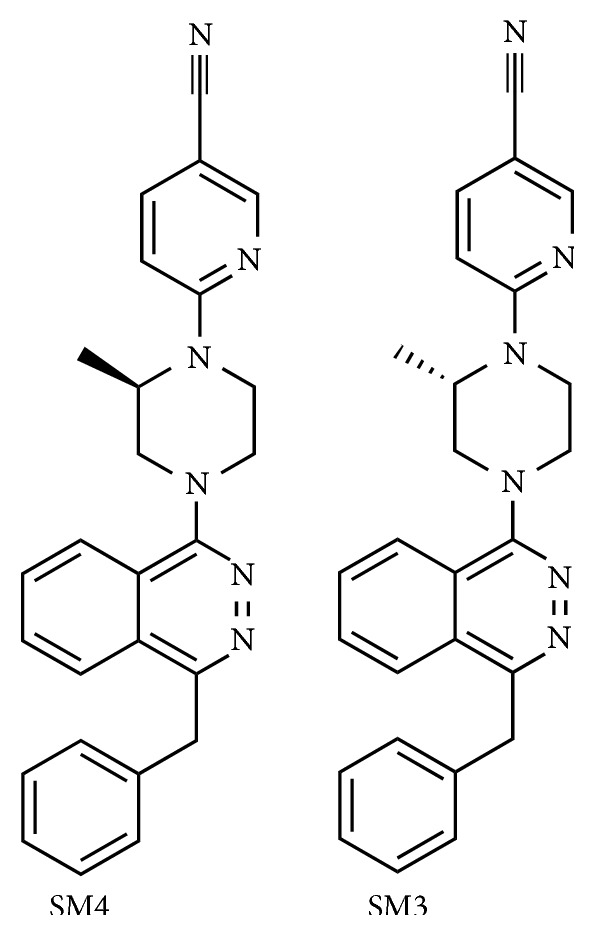
Structures of SM4 and SM3.

Compounds SM4 (SPL inhibitor) and SM3 (the inactive enantiomer) (Figure 1) were prepared as previously described [41] and as shown in Scheme 1. Briefly, the substitution of the chlorine of the commercially available 1-benzyl-4-chlorophthalazine (1) with (*R*)-methylpiperazine or (*S*)-methylpiperazine followed by a second substitution of the chlorine of 6-chloronicotinonitrile with compound 2 or 3 gives us the desired compounds SM4 and SM3. The ^1^H NMR of compounds 2, 3, SM4, and SM3 were identical to those reported previously [41].

### 2.3. Cell Treatment and Viability

Human primary FLS were isolated from articular synovia of donors with RA (RAFLS) or without history of arthritis (normal FLS). Patients from whom synovial specimens were obtained were diagnosed based on the criteria developed by the American College of Rheumatology Diagnostic Subcommittee for RA [42] and underwent arthroplasty. FLS were isolated by sequential enzymatic digestion as described previously [43]. Briefly, FLS were released by sequential enzymatic digestion with 1 mg/mL pronase for 1 h, followed by 6 h with 2 mg/mL collagenase at 37°C in DMEM supplemented with 10% FBS, 1% sodium pyruvate, 100 U/mL penicillin, and 100 mg/mL streptomycin. Released cells were incubated for 1 h at 37°C in tissue culture flasks allowing the adherence of nonfibroblastic cells possibly present in the synovial preparation. The cells were cultured in DMEM supplemented with 10% FBS and antibiotics at 37°C in a humidified atmosphere of 5% CO_2_ and 95% air. Semiconfluent cells were starved with serum-free medium for 24 h before treatment. At the moment of cell treatment, the culture medium was replaced with fresh serum-free medium containing various concentrations of the tested compounds as indicated below. Cells were used between passages 3 and 9. Propidium iodide (PI) was used to evaluate the viability of RAFLS by flow cytometry. Cells were detached using Accutase cell detachment solution and incubated with PI (5 mg/mL). PI negative RAFLS were considered viable.

### 2.4. IL-8 and MCP-1 ELISA

FLS (5 × 10^4^ cells/well) were plated in 24-well plates and serum starved for 24 h prior to stimulation with 200 *μ*M CoCl_2_ or 5 *μ*M S1P for an additional 24 h. Where indicated, cells were pretreated for 30 min with 5 *μ*M of the selective S1P_2_ receptor antagonist JTE-013 and/or selective S1P_3_ receptor antagonist CAY10444, prior to stimulation with CoCl_2_ or S1P. To evaluate the effect of SPL inhibition on CoCl_2_-mediated chemokine secretion, cells were treated with the SPL inhibitor SM4 (or the inactive enantiomer SM3) for 24 h in the absence or the presence of CoCl_2_ and/or sphingosine. Cell culture supernatants were collected and stored at −80°C until the ELISAs were performed. IL-8 and MCP-1 in all samples were monitored in triplicate, according to the manufacturer's protocol. Optical densities were determined using a SoftMaxPro40 plate reader at 450 nm. The results were compared with a standard curve that was generated using known concentrations (pg/mL) of the chemokines. The detection limit of IL-8 and MCP-1 ELISA was 12.5 pg/mL and 15.625 pg/mL, respectively. Data are expressed either as pg/mL or as the percentage of chemokines secreted relative to the appropriate controls.

### 2.5. Quantitative Real-Time PCR

FLS (5 × 10^5^ cells) were plated in 6-well plates and serum starved for 24 h prior to stimulation with or without 200 *μ*M CoCl_2_ in serum-free medium for various times. Total cellular RNA was extracted using TRIzol reagent according to the manufacturer's instructions. RNA (1 *μ*g) was reverse-transcribed using random priming and the Superscript II Reverse Transcriptase system. Real-time PCR was performed to assess the expression of SGPP1, SGPP2, and SPL and their regulation by CoCl_2_. The following sets of primers were used: SGPP1 forward (5′-GCCGCTGGCAGTACCCT-3′) and reverse (5′-AATAGAGTGCATTCCCATGTAAATTCT-3′); SGPP2 forward (5′-TTCAGAACATCCCACCACTCACCA-3′) and reverse (5′-TTCCTGGTGACCACCTTGAACCAT-3′); and SPL forward (5′-GCCAGAGAGTTTATGGTCAAGGTT-3′) and reverse (5′-CAACTTGTCTTGAATCTTACGACCAA-3′). The ribosomal protein RPLP0 mRNA was used as an internal PCR control. RPLP0 primer sequences were as follows: forward (5′-GTTGTAGATGCTGCC-ATTG-3′) and reverse (5′-CCATGTGAAGTCACTGTGC-3′). Amplicon expression in each sample was normalized to its RPLP0 content. The thermal cycling conditions were as follows: 95°C (initial denaturation, 3 min) followed by 40 cycles of 95°C (denaturation, 15 sec), 54°C (annealing, 20 sec), and 72°C (extension, 20 sec).

### 2.6. Western Blot

Cells were exposed to 200 *μ*M CoCl_2_ for various times (0–48 h) and lysed in boiling sample buffer [50 mM Tris/HCL (pH 6.8), 10% (v/v) glycerol, 50 mM DTT, and 4% (v/v) SDS] for 7–10 min. Equal amounts of protein were separated by 10% SDS-polyacrylamide gel electrophoresis and transferred to methanol-soaked Immobilon PVDF membranes (Millipore Corporation, Bedford, MA, USA). Primary antibody incubation was performed either overnight at 4°C (anti-SGPPL, SPL) or 1 h at 37°C (anti-PI3 kinase p85). The membranes were then washed three times and incubated with appropriate horseradish peroxidase-conjugated secondary antibodies at room temperature for 1 h. Membranes were washed three times and antibody-antigen complexes were revealed using Western Lightening ECL^**+**^ according to the manufacturer's instructions (Perkin Elmer Life Sciences, Woodbridge, ON, Canada).

### 2.7. S1P ELISA

FLS from 2 normal and 2 RA donors were cultured up to 80–85% confluence in 75 cm^2^ flasks and serum starved for 24 h. Cells were lysed in 400 *μ*L of lysis buffer provided with the S1P ELISA kit. Protein concentration was measured by the BCA method and S1P in cell lysates (1 : 10 in delipidated human serum) was monitored according to the manufacturer's instructions.

### 2.8. Cytokine/Chemokine Profiling Analysis

RAFLS were treated with the SPL inhibitor SM4 for 24 h in the absence/presence of CoCl_2_ and sphingosine. Cell culture supernatants were collected and stored at −80°C until the Proteome Profiler Human Cytokine Array (panel A) was performed.

### 2.9. Statistical Analysis

Unless otherwise stated, experiments were performed three times for each donor and results presented are expressed as mean ± SE or as representative studies. All statistical analyses were performed using Prism 4.0 software. Statistical significance of the difference between samples of two different treatments was determined by *t*-test (two-tailed *p* value). For multiple comparisons, statistical significance was determined by one-way ANOVA, Dunnett's multiple comparison test. *p* values less than 0.05 were considered statistically significant.

## 3. Results

### 3.1. Chemokine Secretion by Normal FLS and RAFLS in Response to Hypoxic Stress

**Figure 2:**
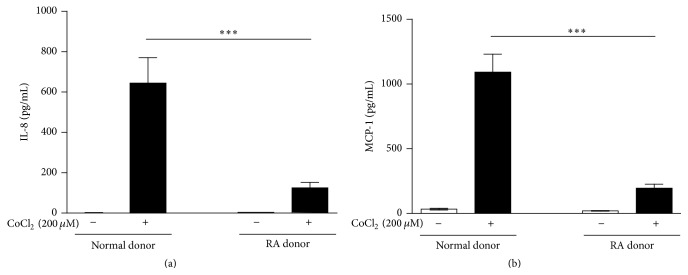
Effect of CoCl_2_ stimulation on IL-8 and MCP-1 secretion in normal FLS and RAFLS. Human primary FLS from normal (*n* = 4) and RA (*n* = 4) donors were incubated with 200 *μ*M CoCl_2_. The amounts of IL-8 (a) and MCP-1 (b) released in the supernatants were monitored 24 h after stimulation. The data are the means ± SE from four experiments (4 different donors) performed in triplicate (3 independent experiments). For statistical comparative analyses, we compared RA to normal FLS treated with CoCl_2_. ^*∗∗∗*^ *p* < 0.001.

To mimic hypoxia, FLS were incubated with CoCl_2_, a chemical inducer of hypoxia-inducible factor-1 (HIF-1) [44]. The effect of chemical hypoxia on chemokine synthesis was assessed using ELISA assays and CoCl_2_-dependent secretion of IL-8 and MCP-1 by normal FLS and RAFLS was compared (Figure 2). Small amounts of IL-8 (<3 pg/mL) (Figure 2(a)) and MCP-1 (<35 pg/mL) (Figure 2(b)) were produced by both normal FLS and RAFLS cultured under normoxic conditions. When incubated with CoCl_2_, normal FLS released significantly larger amounts of IL-8 (644.3 ± 125.9 pg/mL) and MCP-1 (1092 ± 138.6) than RAFLS with similar passage number (125.7 ± 26.5 pg/mL for IL-8 and 195.3 ± 31.9 for MCP-1) (*p* < 0.001). In both control FLS and RAFLS there was a similar trend of decreased synthesis of IL-8 and MCP-1 in response to CoCl_2_ with increased number of cell passages (data not show).

### 3.2. S1P Receptor(s) Dependency of Chemokine Secretion in Normal FLS and RAFLS

**Figure 3:**
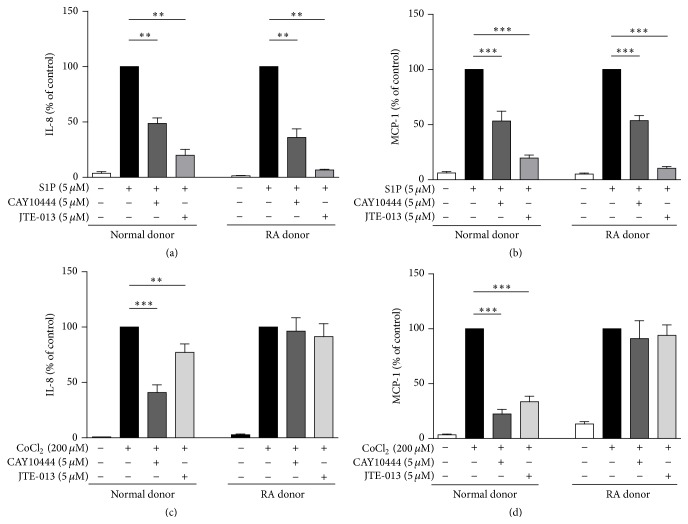
Differential involvement of S1P receptors in S1P- and CoCl_2_-mediated chemokine secretion by normal FLS and RAFLS. Human primary FLS from normal (*n* = 4) and RA (*n* = 4) donors were incubated with 5 *μ*M S1P (a, b) or 200 *μ*M CoCl_2_ (c, d). Where indicated, cells were pretreated with S1P_3_ antagonist CAY10444 (5 *μ*M) or S1P_2_ antagonist JTE-013 (5 *μ*M) for 30 min before stimulation with S1P or CoCl_2_. The amounts of chemokines released in the supernatants were monitored after 24 h. Data are expressed as percentage of chemokine production induced by S1P (a, b) or CoCl_2_ (c, d). The data are the means ± SE from four experiments (4 different donors) performed in triplicate (3 independent experiments). For statistical comparative analyses, the samples stimulated with S1P (a, b) or CoCl_2_ (c, d) were compared to those stimulated in the presence of CAY10444 or JTE-013, respectively. ^*∗∗*^ *p* < 0.01; ^*∗∗∗*^ *p* < 0.001.

S1P regulates a variety of cellular processes through binding to G protein-coupled receptors [45]. We previously reported a role for S1P_2_ and S1P_3_ in S1P-mediated IL-8 secretion in RAFLS [33]. As expected, the addition of S1P to normal FLS and RAFLS stimulated the secretion of IL-8 and MCP-1. The amounts of IL-8 and MCP-1 released by normal FLS and RAFLS in response to S1P were not statistically different (149.0 ± 28.62 versus 126.9 ± 14.3 pg/mL for IL-8 (*p* = 0.47) and 800.3 ± 116.4 pg/mL versus 546.5 ± 69.42 pg/mL for MCP-1 (*p* = 0.10)). Under these conditions the S1P_3_ antagonist CAY10444 and the S1P_2_ antagonist JTE-013 significantly decreased S1P-induced IL-8 by 51.3 ± 5.0%  (*p* < 0.01) and 80.1 ± 5.4%  (*p* < 0.01) in normal FLS and by 63.9 ± 7.8%  (*p* < 0.01) and 93.3 ± 0.6%  (*p* < 0.01) in RAFLS, respectively (Figure 3(a)). CAY10444 and JTE-013 also reduced S1P-mediated MCP-1 secretion by 46.7 ± 8.9%  (*p* < 0.001) and 80.3 ± 2.7%  (*p* < 0.001) in normal FLS and that of RAFLS by 46.4 ± 4.5%  (*p* < 0.001) and 89.6 ± 1.6%  (*p* < 0.001), respectively (Figure 3(b)). Similarly, the incubation in normal FLS with CAY10444 and JTE-013 in combination with CoCl_2_ reduced IL-8 secretion by 59.0 ± 6.8%  (*p* < 0.001) and 22.0 ± 7.5%  (*p* < 0.01) and that of MCP-1 by 77.6 ± 4.2%  (*p* < 0.001) and 66.4 ± 5.0%  (*p* < 0.001), respectively (Figures 3(c) and 3(d)). In contrast, the production of chemokines by RAFLS incubated with CoCl_2_ was not inhibited by the S1P_3_ or the S1P_2_ receptor antagonist (Figures 3(c) and 3(d)). The percentage of PI positive cells treated with 200 *μ*M CoCl_2_ together with 5 *μ*M CAY10444 and 5 *μ*M JTE-013 for 24 h was identical to that of untreated cells (1.25 ± 0.15% versus 1.4 ± 0.3% for normal FLS treated with CoCl_2_/CAY10444 versus untreated, 1.15 ± 0.15% versus 1.4 ± 0.3% for normal FLS treated with CoCl_2_/JTE-013 versus untreated; 1.2 ± 0% versus 1.0 ± 0.1% for RAFLS treated with CoCl_2_/CAY10444 versus untreated, and 1.15 ± 0.25% versus 1.0 ± 0.1% for RAFLS treated with CoCl_2_/JTE-013 versus untreated), indicating that inhibition of chemokine synthesis was not mediated by a cytotoxic effect of these compounds.

### 3.3. Intracellular Levels of S1P in Normal FLS and RAFLS

The response of normal FLS and RAFLS to exogenously added S1P and inhibition of chemokine secretion by the S1P_3_ and S1P_2_ receptor antagonists provide evidence for functional S1P receptors in both types of FLS. On the other hand, inhibition of CoCl_2_-dependent chemokine synthesis by the S1P antagonists in normal FLS but not in RAFLS points toward alteration of an autocrine positive feedback loop driven by S1P. This could be due to impaired steady levels of intracellular S1P and/or export outside cells. To gain insight into the possible mechanisms we monitored the intracellular levels of S1P in normal FLS and in RAFLS. As shown in Table 1 the basal level of intracellular S1P was more elevated in normal FLS as compared to RAFLS.

### 3.4. Regulation of the Expression of the S1P Degradation Enzymes by CoCl_2_ in Normal FLS and RAFLS

**Figure 4:**
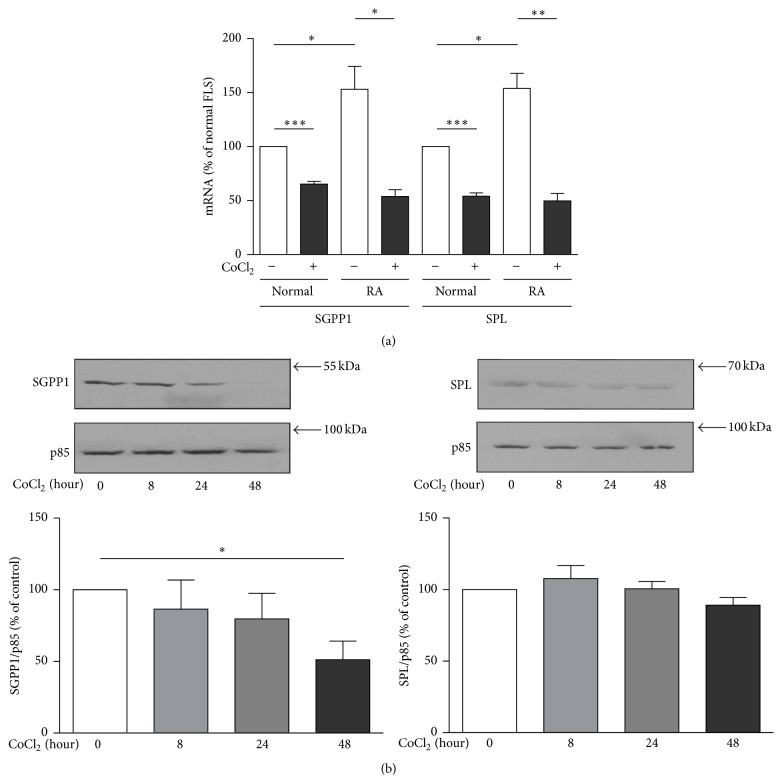
Differential expression of SGPP1 and SPL in normal FLS and RAFLS. Human primary FLS from normal (*n* = 4) and RA (*n* = 4) donors were incubated with or without 200 *μ*M CoCl_2_ for 24 h. Total RNA was extracted for quantitative PCR analyses and RPLP0 was used as an internal control and data normalized to that of normal FLS (a). The data are the means ± SE from four experiments (4 different donors) performed in triplicate (3 independent experiments). For statistical analyses, we compared the cells stimulated with CoCl_2_ to those without CoCl_2_, or normal FLS to RAFLS. ^*∗*^ *p* < 0.05; ^*∗∗*^ *p* < 0.01; ^*∗∗∗*^ *p* < 0.001. Human primary FLS from RA patients (*n* = 3) were incubated with 200 *μ*M CoCl_2_ for up to 48 h (b). Proteins from whole cell lysates were prepared for Western blot. Total PI3-kinase p85 subunit was used as a control for protein loading. Data presented are from a representative blot (upper panel) or the means ± SE from three experiments (lower panel). For statistical comparative analyses, the samples stimulated with CoCl_2_ at 0 h were compared to those treated for indicated times. ^*∗*^ *p* < 0.05.

Decreased steady state levels of intracellular S1P in RAFLS could be due to altered production of S1P by SphKs, increased degradation by S1P phosphatases (SGPP1 and SGPP2) or S1P lyase (SPL), and/or a combination of the two mechanisms. In this study we focussed on the impact of CoCl_2_ on S1P phosphatases and SPL gene/protein expression in normal FLS and in RAFLS. Quantitative real-time PCR (qPCR) analyses highlighted the expression of SGPP1 and SPL mRNA in cells (Figure 4(a)). SGPP2 mRNA was not detected with the primers we designed for this study (data not shown). As shown in Figure 4(a), SGPP1 and SPL mRNA were ~1.5- and 1.53-fold more abundant in RAFLS than in normal FLS (*p* < 0.05). Moreover, incubation with CoCl_2_ decreased SGPP1 mRNA levels by 34.7 ± 2.5%  (*p* < 0.001) and 64.8 ± 6.2%  (*p* < 0.05) and those of SPL mRNA by 45.9 ± 3.0%  (*p* < 0.001) and 67.7 ± 6.9%  (*p* < 0.01) in normal FLS and RAFLS, respectively (Figure 4(a)). Decreased expression of SGPP1 was confirmed at the protein level in RAFLS with a 48.8 ± 13.1% decrease in SGPP1 protein (*p* < 0.05) after treatment with CoCl_2_ for 48 h (Figure 4(b)). SPL protein levels in RALFS were not significantly reduced by CoCl_2_ as estimated by immunoblotting (Figure 4(b)).

### 3.5. Effect of SPL on CoCl_2_-Mediated Chemokine Secretion by Normal FLS and RAFLS

**Figure 5:**
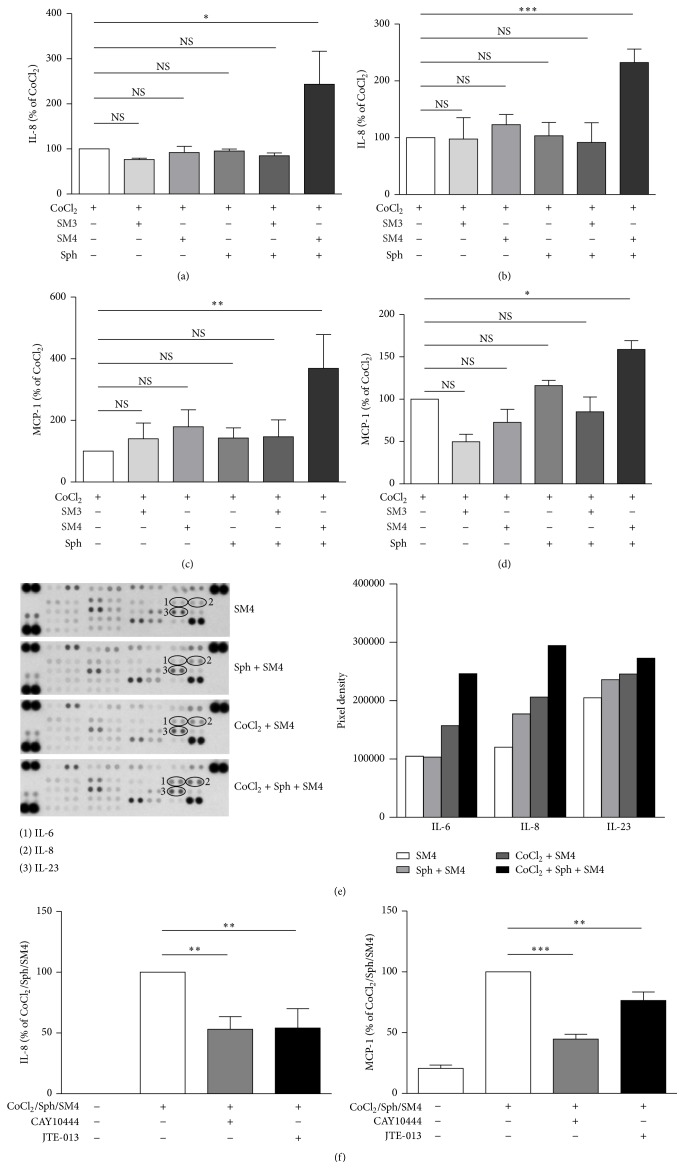
Impact of SPL inhibition on CoCl_2_-mediated chemokine/cytokine secretion in normal FLS and RAFLS. Human primary FLS from normal (a, c) and RA (b, d, e, f) donors were incubated with 200 *μ*M CoCl_2_ in the presence of SPL inhibitor SM4 (3 *μ*M) or the inactive analog SM3 (3 *μ*M) and sphingosine (1 *μ*M) for 24 h. Where indicated, cells were pretreated with S1P_3_ antagonist CAY10444 (5 *μ*M) or S1P_2_ antagonist JTE-013 (5 *μ*M) for 30 min before stimulation with CoCl_2_ in combination with sphingosine (Sph), SM4, or SM3. The data are the means ± SE from three experiments. For statistical comparative analyses, chemokine levels in the samples stimulated with CoCl_2_ were compared to that of other samples (a–d) or chemokines produced by cells stimulated with CoCl_2_ in combination with Sph and SM4 were compared to those produced by cells incubated with the S1P receptor antagonists prior to cell stimulation (f). ^*∗*^ *p* < 0.05; ^*∗∗*^ *p* < 0.01; ^*∗∗∗*^ *p* < 0.001. Cytokine/chemokine secretion in RAFLS supernatants was analyzed using Proteome Profiler Human Cytokine Array panel A (e). Circled pairs of duplicate spots represent one cytokine/chemokine.

To determine whether the levels of intracellular S1P in FLS may impact its transport outside cells and access to its cognate receptors for autocrine signalling we incubated the cells with CoCl_2_ in the presence or absence of a SPL inhibitor [46, 47]. When normal FLS and RAFLS were incubated with CoCl_2_ in combination with increasing concentrations of the SPL inhibitor SM4 there was a trend towards increased secretion of IL-8 and MCP-1 (Figure 5 and data not shown). However, even with 3 *μ*M SM4, the highest concentration tested, the increase in chemokine synthesis was not significant compared to cells treated with CoCl_2_ alone (data not shown). Since the addition of sphingosine to cell line or primary cell cultures has been shown to provide a source of intracellular S1P that is susceptible to degradation by SPL [46, 47], we evaluated the impact of exogenously added sphingosine in combination with the SPL inhibitor on CoCl_2_-mediated chemokine synthesis. Figure 5 shows that the inhibition of SPL in the presence of sphingosine significantly increased CoCl_2_-induced chemokine secretion in RAFLS (Figures 5(b) and 5(d)) and in normal FLS as well (Figures 5(a) and 5(c)). In RAFLS SM4 increased the secretion of IL-8 and MCP-1 by 232 ± 23.8%  (*p* < 0.001) and 158.7 ± 10.7%  (*p* < 0.05), respectively, while in normal FLS SM4 increased IL-8 and MCP-1 secretion by 243.3 ± 73.4%  (*p* < 0.05) and 368.5 ± 109.7%  (*p* < 0.01), respectively. No significant increase in chemokine synthesis was observed when cells were incubated with CoCl_2_ in the presence of sphingosine without the SPL inhibitor, with the SPL inhibitor but without sphingosine, or with the inactive enantiomer SM3. The Proteome Profiler Antibody Array confirmed in RAFLS that inhibition of SPL in combination with sphingosine increases CoCl_2_-mediated IL-8 secretion and possibly that of other cytokines such as IL-6 and IL-23 (Figure 5(e)). When RAFLS were treated with the SPL inhibitor in the presence of sphingosine (Figure 5(f)), CoCl_2_-mediated secretion of IL-8 and MCP-1 becomes sensitive to inhibition by the S1P_3_ receptor antagonist CAY10444 (46.9 ± 10.3% and 55.3 ± 4.0% decrease, *p* < 0.01, resp.) and the S1P_2_ receptor antagonist JTE-013 (45.9 ± 15.9% and 23.5 ± 7.0% decrease, *p* < 0.01, resp.).

## 4. Discussion

FLS are key effector cells in RA. They spread arthritis to unaffected joints [48] and their altered phenotypes in RA have been associated with changes in signalling cascades, apoptotic responses, and the expression of adhesion molecules as well as matrix-degrading enzymes [49, 50]. The cell microenvironment plays an essential role in determining cell phenotype and phenotypic and metabolic characterization of those changes will further our understanding of the pathogenesis of RA. Herein, we report novel characteristics of RAFLS that distinguish these cells from their normal counterparts: (1) RAFLS are less prone to release IL-8 and MCP-1 in response to the hypoxia mimetic CoCl_2_; (2) CoCl_2_-mediated chemokine production is, at least in part, due to autocrine activation of S1P receptors in control FLS but not in RAFLS; (3) expression of SGPP1 and SPL mRNA is elevated whereas intracellular levels of S1P are reduced in RAFLS when compared to normal FLS; (4) whereas CoCl_2_ reduces SGPP1 mRNA and protein expression, the combination of the hypoxic-like stress, sphingosine, and inhibition of SPL is required to enhance chemokine/cytokine synthesis and to restore a positive autocrine feedback loop of chemokine synthesis depending on S1P receptor activation in RAFLS. The data suggest that sphingolipid metabolism is altered in RAFLS collected from patients with advanced RA.

Hypoxia was reported to potentiate the expression of inflammatory cytokines, MMPs, and VEGF in RAFLS stimulated with TLR ligands [51]. Moreover, hypoxia has been shown to induce the expression of IL-8 mRNA in RAFLS [24]. In agreement with those findings we report that the hypoxia mimetic agent CoCl_2_ stimulated IL-8 and MCP-1 production in normal FLS and RAFLS. Surprisingly, the amounts of IL-8 and MCP-1 released by RAFLS incubated with CoCl_2_ were less than those produced by normal FLS, indicative of altered molecular pathways regulating chemokine synthesis in RAFLS. RAFLS phenotypic changes are possibility related to genetic/epigenetic determinants and genetic mutation due to chronic exposure to a hypoxic inflammatory environment [52]. Indeed, the expression of many genes involved in immune and inflammatory function is differently regulated by hypoxia in normal FLS and RAFLS [53]. The proinflammatory chemokines/cytokines MCP-2, MIP-2*α*, MIP-2*β*, and IL-12A for instance are downregulated whereas the anti-inflammatory mediators CD300a and AMPD3 are upregulated by hypoxia in RAFLS [53].

Upregulation of SphK1 expression and activation by hypoxia has been linked to increases in intracellular and extracellular S1P levels [54]. Previous studies have highlighted the expression of S1P_1_, S1P_2_, and S1P_3_ receptors in RAFLS [33, 34]. High expression of S1PR_1_ in RA synovial tissue was observed in the synovial lining, vascular endothelial cells, and mononuclear cells when compared to osteoarthritis and normal synovial tissues [34]. In vitro S1P induces RAFLS migration, expression of cytokines/chemokines and COX-2, prostaglandin synthesis, and cell proliferation and survival [33, 34]. S1P receptors expressed by RAFLS have redundant functions. In a wound-closing assay S1P induced RAFLS migration through S1P_1_ and S1P_3_ receptors [33]. On the other hand, S1P stimulated the secretion of numerous cytokines/chemokines (IL-8, IL-6, MCP-1, and RANTES) through S1P_2_ and S1P_3_ receptors. In the present study we provide evidence that the mechanism by which CoCl_2_ induces the secretion of chemokines is, at least in part, through autocrine activation of S1P_2_ and S1P_3_ receptors in normal FLS. Although RAFLS express functional S1P_2_ and S1P_3_ receptors, CoCl_2_-mediated chemokine synthesis was not reduced by S1P receptor antagonists. This was related to low levels of intracellular S1P in RAFLS since incubation of cells with an inhibitor of SPL and sphingosine, a condition that has been shown to increase intracellular amounts of S1P and its release by various cells [46, 47], restores autocrine signalling through S1P_2_ and S1P_3_ receptors in RAFLS stimulated with CoCl_2_.

S1P synthesis requires the concerted action of ceramidase and sphingosine kinases and once formed, S1P is either metabolized to hexadecenal and ethanolamine phosphate by SPL or recycled to sphingosine by S1P phosphatases [30]. Upregulation of SGPP2 has been detected in samples of skin lesions from patients with psoriasis, a chronic inflammatory skin disease [55]. Other studies investigating sphingolipid metabolism have shown that oxygen deprivation in microendothelial cells resulted in reduced SPL activity [56] and that adipocytes respond to hypoxia by downregulating SPL expression [57]. In this study we provide evidence for increased expression of SGPP1 and SPL mRNA in RAFLS, suggesting that the lower level of intracellular S1P in these cells is possibly driven by a hypercatabolic state. Targeting S1P_1_ receptor with a selective antagonist [38] or with the sphingosine analogue FTY720 [58, 59] and pharmacological inhibition of SPL in mice [60], all decreased the development of collagen-induced arthritis (CIA). The anti-inflammatory properties of these compounds are associated with abnormal B and T cell maturation and lymphocyte egress from lymphoid organs due to local S1P gradient breakdown or S1P_1_ receptor degradation [38, 61, 62]. Whereas inhibition of SPL may have a beneficial effect through targeting lymphocyte trafficking from lymphoid organs, we suggest that inhibition of SPL may have adverse inflammatory effects by increasing the steady state levels of intracellular S1P, S1P export, and synthesis of proinflammatory chemokines/cytokines through autocrine/paracrine activation of S1P_2_ and S1P_3_ receptors. Allende et al. recently reported that SPL deficiency in mice promotes an inflammatory response [63].

A few studies have evaluated S1P levels and S1P metabolizing enzymes in RA synovial biopsy. For example, expression of SphK2 and elevated levels of S1P were detected in the synovium and synovial fluids of RA patients [34–36]. Animal models have been used to evaluate the role of S1P in inflammatory arthritis. In the CIA model, administration of a nonspecific inhibitor of SphKs or of a siRNA to silence SphK1 markedly suppressed cartilage and bone erosion, synovial hyperplasia, and leukocyte infiltration into the joint compartments [36]. While SphK1 activity is proinflammatory, SphK2 has an opposite role since the silencing of this enzyme in mice promotes CIA-mediated synovitis [64]. However, depending on the animal models of arthritis employed, studies with KO mice have produced conflicting information. Whereas SphK1 deficiency has been reported to reduce synovial inflammation and bone erosions in human TNF-*α* transgenic mice, which spontaneously develop inflammatory arthritis [37], SphK2 deficiency has no impact on disease severity and progression [65]. Our preliminary data suggest that CoCl_2_ induces SphK1 expression in normal FLS whereas SphK1 seems to be less prone to upregulation by CoCl_2_ in RAFLS (data not shown). Further characterization is underway to determine whether altered expression and/or activation of Sphks contribute to reduced steady state levels of intracellular S1P in RAFLS.

In summary, the results of this study suggest that the sphingolipid metabolism involved in the production and/or release of S1P under hypoxic-like conditions is altered in RAFLS. Decreased steady state levels of intracellular S1P in RAFLS were associated with reduced production of chemokine/cytokine and autocrine activation of S1P_2_ and S1P_3_ receptors in response to chemical hypoxia. Our data provide new insights into the mechanisms that may regulate inflammation and possibly joint destruction in advanced cases of RA.

## Figures and Tables

**Table 1:** S1P content in normal FLS and RAFLS.

	S1P content (pmol/mg of protein)
Normal FLS	
Donor #1 (S3618)	64.5 ± 1.5
Donor #2 (S3739)	273.0 ± 21
RAFLS	
Donor #1 (37158A1-S)	19.5 ± 1.5
Donor #2 (87546A1-S)	22.5 ± 1.5

Cell lysates from human primary FLS of normal (*n* = 2) and RA (*n* = 2) donors were prepared. S1P content in cell lysates (50 *μ*g protein) was measured using the S1P assay kit from Echelon Inc. according to the manufacturer's instruction.
